# Analysis and reporting of stratified cluster randomized trials—a systematic survey

**DOI:** 10.1186/s13063-020-04850-w

**Published:** 2020-11-17

**Authors:** Sayem Borhan, Alexandra Papaioannou, Jinhui Ma, Jonathan Adachi, Lehana Thabane

**Affiliations:** 1 Department of Health Research Methods, Evidence and Impact, McMaster University, Hamilton, ON Canada; 2 Biostatistics Unit, Research Institute of St Joseph’s Healthcare, Hamilton, ON Canada; 3 GERAS Centre, Hamilton Health Sciences, Hamilton, ON Canada; 4 Department of Medicine, McMaster University, Hamilton, ON Canada; 5 Departments of Pediatrics and Anesthesia, McMaster University, Hamilton, ON Canada

**Keywords:** Stratification, Cluster randomized trial, Systematic survey, Stratified cluster, Randomized trial

## Abstract

**Background:**

In order to correctly assess the effect of intervention from stratified cluster randomized trials (CRTs), it is necessary to adjust for both clustering and stratification, as failure to do so leads to misleading conclusions about the intervention effect. We have conducted a systematic survey to examine the current practices about analysis and reporting of stratified CRTs.

**Method:**

We used the search terms to identify the stratified CRTs from MEDLINE since the inception to July 2019. In phase 1, we screened the title and abstract for English-only studies and selected, including the main results paper of the identified protocols, for the next phase. In phase 2, we screened the full text and selected studies for data abstraction. The data abstraction form was piloted and developed using the REDCap. We abstracted data on multiple design and methodological aspects of the study including whether the primary method adjusted for both clustering and stratification, reporting of sample size, randomization, and results.

**Results:**

We screened 2686 studies in the phase 1 and selected 286 studies for phase 2—among them 185 studies were selected for data abstraction. Most of the selected studies were two-arm 140/185 (76%) and parallel-group 165/185 (89%) trials. Among these 185 studies, 27 (15%) of them did not provide any sample size or power calculation, while 105 (57%) studies did not mention any method used for randomization within each stratum. Further, 43 (23%) and 150 (81%) of 185 studies did not provide the definition of all the strata, while more than 60% of the studies did not include all the stratification variable(s) in the flow chart or baseline characteristics table. More than half 114/185 (62%) of the studies did not adjust the primary method for both clustering and stratification.

**Conclusion:**

Stratification helps to achieve the balance among intervention groups. However, to correctly assess the intervention effect from stratified CRTs, it is important to adjust the primary analysis for both stratification and clustering. There are significant deficiencies in the reporting of methodological aspects of stratified CRTs, which require substantial improvements in several areas including definition of strata, inclusion of stratification variable(s) in the flow chart or baseline characteristics table, and reporting the stratum-specific number of clusters and individuals in the intervention groups.

**Supplementary information:**

**Supplementary information** accompanies this paper at 10.1186/s13063-020-04850-w.

## Background

The random allocation of intact group of subjects—termed as clusters—into intervention groups is commonly known as cluster randomized trials (CRTs) [1]. The number of adopting CRTs to assess the effect of intervention is increasing [2]. The type of clusters can be diverse such as geographical areas [3], healthcare districts [4], and schools [5]. There are several types of experimental designs that are used to allocate clusters into intervention arms, including completely randomized, stratified, and matched-pair design. Clusters are randomly allocated to intervention groups within each stratum in a stratified design, which is suitable for small number of clusters [6].

The potential degree of similarity among the outcomes from the same cluster, measured through intra-cluster correlation coefficient (ICC), should be taken into account to assess the intervention effect from cluster randomized trials [1]. The failure to account for this correlation may yield a false positive result [1, 7]. Scientists have developed and recommended statistical methods that can be used to examine the intervention effect, while taking the clustering [1] into account. In addition, in the case of a stratified design, the statistical methods need to adjust for stratification [1]. It has been shown in the literature that variables used in the randomization process should be adjusted for in the analysis [8–13]. The absence of such adjustment in the analysis can yield large *p* values and wider confidence intervals, which could potentially lead to a misleading conclusion that the intervention has no effect [14]. Borhan et al. [15] empirically compared the methods for analyzing continuous data from stratified CRTs and reported that confidence intervals were wider when not adjusted for stratification, compared to when adjusted for stratification for the corresponding method.

Thus, to correctly assess the effect of intervention, it is important to adjust for stratification variables as it will yield correct *p* values and confidence intervals. Kahan and Morris [14] conducted a small-scale review on randomized trials on individuals and reported that only 26% of the studies adjusted for the balancing factors in their primary analysis. On the other hand, systematic reviews of CRTs demonstrated that there are significant deficiencies in the design, analysis, and reporting of CRTs [2, 16–25]. For example, in their systematic review of CRTs in primary healthcare, Eldridge et al. [21] found that 20% of the studies adjusted for clustering in sample size calculation, while 59% adjusted the analyses for clustering. Others have argued that inclusion of statistician in the research team can significantly improve the quality of reporting and analysis of CRTs [24]. Further, CONSORT statement has been extended for CRTs to guide the researchers about reporting of CRTs [26]. However, we have limited or no knowledge of how often the assessment of intervention effect from the stratified CRTs adjusted for clustering and stratification occurred.

In this study, we conducted a systematic survey to examine the analysis and reporting of stratified CRTs, which covered several methodological and reporting aspects including how often the primary method to examine the effect of intervention adjusted for both clustering and stratification, as well as whether the reporting of sample size calculations, randomization, and stratification was adequate.

## Method

In this systematic survey, we identified the stratified cluster randomized trials and abstracted data on multiple study characteristics, including sample size estimation, randomization, analysis, and reporting.

### Search strategy and study selection

We added the term “strati*” with the search terms (Table 1) suggested by Taljaard et al. [27] to identify the stratified cluster randomized trials from MEDLINE since the inception to July 2019. First, we performed title and abstract screening and selected the English-only studies, including the main results paper of the identified protocols. In the second phase, we screened the full text of the papers selected in the first phase and selected the studies for data abstraction. We used the protocol papers to identify the published main study results and included those in the study. In the case of multiple articles from the same trial, we included only the main study results. Selection of studies was performed using EndNote X8. PRISMA checklist [28] (Additional file 1) was used for reporting.

**Table 1:** Search terms used to identify studies from MEDLINE since the inception to July 2019

1	randomized controlled trial.pt. (485792)
2	animals/ (6439569)
3	humans/ (17863499)
4	2 not (2 and 3) (4567683)
5	1 not 4 (474298)
6	(clusters*adj2randomi *).tw. (203)
7	((communit*adj2intervention*) or(communit*adj2randomi*)).tw. (7588)
8	groupr*andomi*.tw. (3177)
9	6 or 7 or 8 (10908)
10	intervention?.tw. (861625)
11	cluster analysis/ (59383)
12	health promotion/ (70103)
13	program evaluation/ (59930)
14	health education/ (59114)
15	10 or 11 or 12 or 13 or 14 (1051673)
16	9 or 15 (1053569)
17	16 or 5 (1434924)
18	16 and 5 (92943)
19	strati*.mp. (168454)
20	18 and 19 (2686)

### Data abstraction

A data abstraction form was piloted and developed using REDCap. Data abstraction form includes data on many study characteristics, including country, clinical area, setting of the study, sample size calculation, randomization, analysis of primary outcome, and reporting.

### Outcome and analysis

We abstracted data on several methodological and reporting areas related to stratified CRTs, and descriptive summary, such as *n* (%) or mean (standard deviation [SD]) or median (first quartile [Q1], third quartile [Q3]), was used to analyze the outcomes. There were several outcomes related to the reporting of sample size or power calculation, including whether the sample size or power calculation reported, level of significance and desired power reported, and whether the sample size adjusted for the lost to follow-up. Similarly, we abstracted data on several issues related to randomization, including randomization unit, number and type of stratification variables and strata, and method used for randomization within each stratum. Several outcomes from the method of primary outcome analysis were analyzed, including type of primary outcome, unit of analysis, type of primary analysis, whether the primary method adjusted for stratification or clustering or both, how the primary method adjusted for stratification variables, whether missing data were imputed or sensitivity analysis was performed, and statistical significance of intervention effect (only for 2-arm trials). Moreover, we abstracted data on several outcomes related to reporting, including whether study flow chart or baseline characteristics table included stratification variable(s), numbers of clusters or individuals for each stratum were provided, and the estimated ICC was reported. See the “Results” section for details about the outcomes. All analyses were performed using R.

## Results

**Fig. 1:**
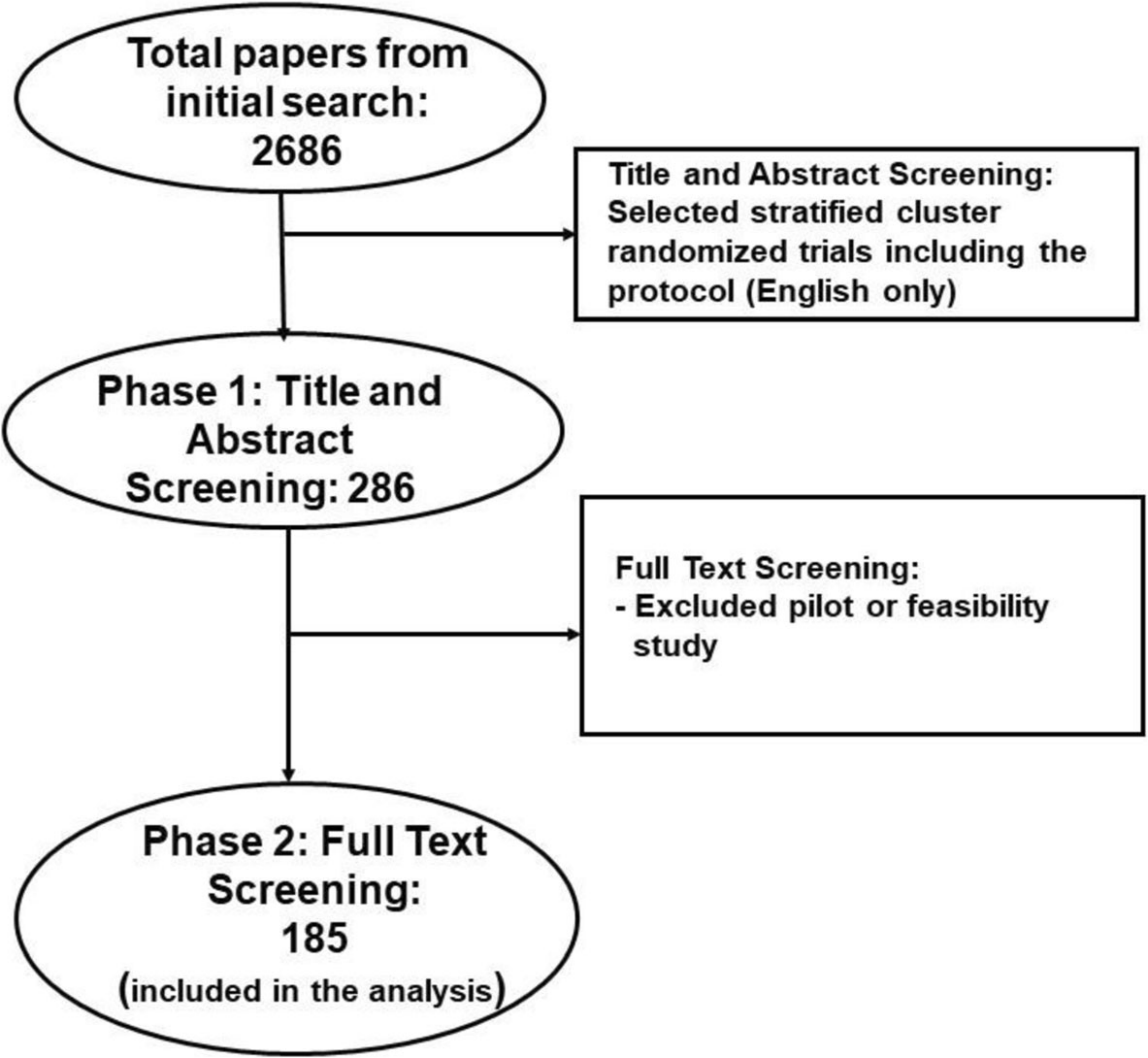
Flow chart of study selection

Our search produced 2686 published reports, and after initial screening followed by assessment of full text, 185 papers (all reporting 185 unique CRTs) were selected for analysis (Fig. 1).

The results of some basic characteristics of the selected studies are provided in Table 2. About 80% of the studies were from 2010 to 2019, while only 7 (4%) studies were from before 2000. Almost half of the studies, 48%, were one centered, and most of the studies (31%) were conducted in the USA or UK (Table 2). Among these studies, 36 (19%) and 27 (15%) of them were focused on interventions related to child development or primary care/general practices, respectively. Also, there were 36 and 38 studies that were school- or general practice-based, respectively (Table 2). In addition, the majority—140 (76%) and 165 (89%)—of the studies were 2-arm and parallel-group trials, respectively (Table 2).

**Table 2:** Results of study characteristics

Characteristics	Number of studies included; ***n*** = 185
**Publication year**; *n* (%)	
Before 2000	7 (4)
Between 2001 and 2010	30 (16)
Between 2011 and 2019	148 (80)
**Center of study**; *n* (%)	
One	89 (48)
Two	44 (24)
Three or more	52 (28)
**Country of the study**; *n* (%)	
UK	32 (17)
USA	26 (14)
Canada	8 (4)
India	7 (4)
Australia	15 (8)
Denmark	7 (4)
Germany	5 (3)
Netherlands	6 (3)
South Africa	8 (4)
Others	71 (39)
**Clinical area**; *n* (%)	
Child development	36 (19)
Primary care	27 (15)
Maternal and child health	16 (9)
HIV	12 (6)
Cancer	9 (5)
Malaria	9 (5)
Cardiovascular	5 (3)
Cognitive and mental health	10 (5)
Others	61 (33)
**Setting of the study**; *n* (%)	
School	36 (19)
General practice/primary care	38 (21)
Community	36 (19)
Hospital	18 (10)
Village	10 (5)
Family	6 (3)
Others	41 (22)
**Design of the study**; *n* (%)	
Parallel	165 (89)
Cross-over	2 (1)
Stepped wedge	3 (2)
Factorial	7 (4)
Matched pair	6 (3)
Split-plot	1 (1)
Zelen design	1 (1)
**Arm of the study**; *n* (%)	
2	140 (76)
3	28 (15)
4	14 (8)
5	2 (1)
6 or more	1 (1)

**Fig. 2:**
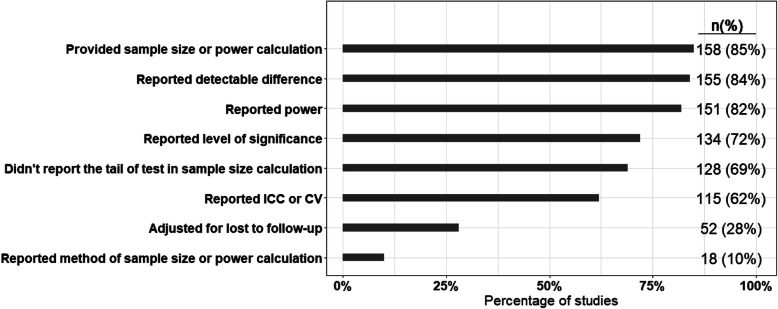
Results of outcomes related to sample size or power calculation among all the studies (*n* = 185)

**Fig. 3:**
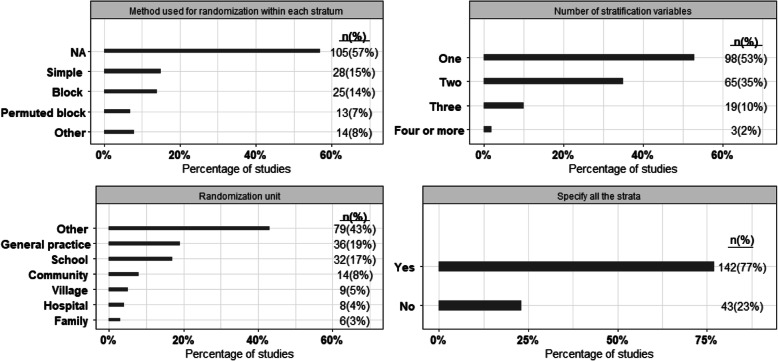
Results of outcomes related to randomization among all the studies (*n* = 185) except type of stratification variables. *NA, not available

One hundred and fifty-eight (85%) out of 185 studies provided sample size or power calculations, while 66% of the studies adjusted for clustering (Fig. 2). While more than 80% of the studies reported the level of significance or desired power in sample size or power calculations, only 10% of the studies reported the method used, and 28% of the studies adjusted for lost to follow-up in sample size calculations (Fig. 2). Akin to the setting of the study, almost similar numbers of studies used school or primary care/general practice as the randomization unit (Fig. 3). Almost half of the studies had one stratification variable, while only 2% of the studies had 4 or more stratification variables. In total, there were 298 stratification variables used across all included trials. About 35% (103/298) stratification variables were based on geographical location, while 17% (51/298) and 15% (46/298) were based on epidemiologic/prognostic factors and cluster size, respectively. More than half of the studies (57%; 105/185) did not provide the method used for randomization, while 23% (43/185) of the studies specified all the strata (Fig. 3).

The results of outcomes related to the primary method of analysis are provided in Table 3. The primary outcome of 83% (152/185) of the studies was continuous or binary. One hundred and forty-three (77%) studies performed individual-level analysis. More than half (52%) of the studies used an intention-to-treat approach as their primary analysis approach, while 43% of the studies did not mention their primary analysis approach (Table 3). Seventy-one (38%) studies reported primary method/effect estimate adjusted for both clustering and stratification. Among the studies that adjusted for stratification, 90% (66/73) of the studies adjusted for stratification by using them as the covariate(s) (Table 3). Thirty-eight (46%; 38/82) of the studies reported their statistically significant intervention effect, among the 2-arm studies that did not adjust their primary method for both clustering and stratification (Table 3).

**Table 3:** Results of outcomes related to analysis method among all the studies (*n* = 185) except for significance of intervention effect

Outcome	***n*** = 185
**Type of primary outcome**; *n* (%)	
Continuous	64 (35)
Binary	88 (48)
Count	28 (15)
Time to event	3 (2)
Other	2 (1)
**Unit of analysis**; *n* (%)	
Cluster-level	28 (15)
Individual-level	143 (77)
Not clear	14 (8)
**Primary approach of analysis**; *n* (%)	
Intention-to-treat	96 (52)
Per-protocol	9 (5)
Not available	80 (43)
**Primary method/reported effect estimate adjusted for clustering or stratification**; *n* (%)	
Clustering and stratification	71 (38)
Clustering only	92 (50)
Stratification only	2 (1)
None	20 (11)
**Type of adjustment for stratification**; *n* (%) [*n* = 73]	
As a covariate	66 (90)
Stratum-specific estimate and then combine	5 (7)
Stratum-specific estimate	2 (3)
**Imputed missing data**; *n* (%)	
No	150 (81)
Yes	35 (19)
**Performed sensitivity analysis**; *n* (%)	
No	127 (69)
Yes	58 (31)
**Intervention effect significant**; *n* (%) [2-arm trials only; *n* = 140]	
No	76 (54)
Yes	64 (46)
**Significance of intervention effect among those adjusted for both clustering and stratification**; *n* (%) [*n* = 58]	
No	32 (55)
Yes	26 (45)
**Significance of intervention effect among those not adjusted for both clustering and stratification**; *n* (%) [*n* = 82]	
No	44 (54)
Yes	38 (46)

**Fig. 4:**
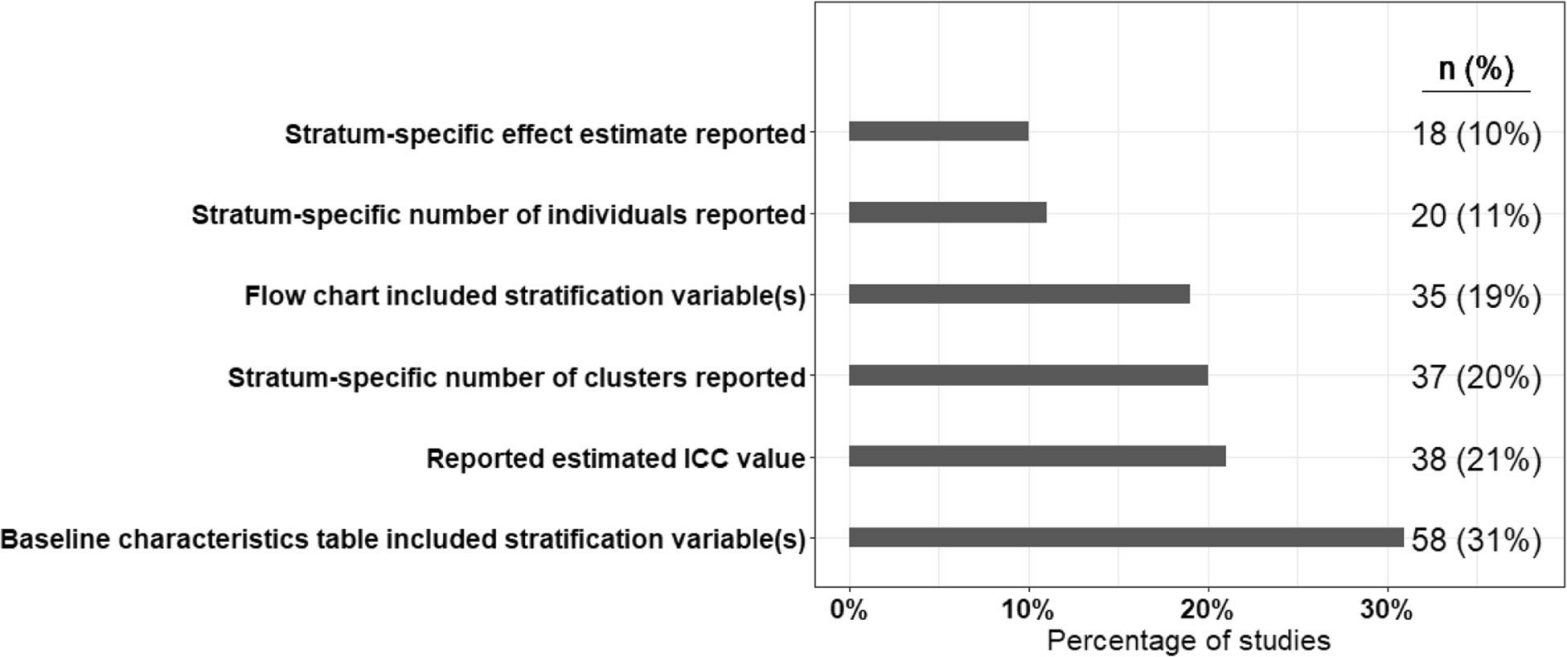
Results of reporting outcomes among all the included studies (*n* = 185)

The results of outcomes pertaining to reporting of outcomes are provided in Fig. 4. Only 19% (35/185) and 31% (58/185) of the studies included stratification variables in the flow chart or baseline characteristics table. Only 10% (18/185) of the studies reported stratum-specific effect estimate.

## Discussion

In this systematic survey, we selected 185 stratified cluster randomized trials from MEDLINE since the inception to July 2019, and found that there were significant deficiencies in the reporting of methodological aspects of stratified CRTs, and only 38% of the studies adjusted the primary method for both clustering and stratification. This result is similar to the findings of Kahan and Morris [14], as they reported 26% of the studies adjusted for balancing or stratification factors.

In order to correctly assess the effect of intervention, it is important to adjust the primary method for stratification variable(s) as well as clustering [14], which was also established from the empirical study of Borhan et al. [15]. From this systematic review, it is evident that this type of adjustment is still scarce as more than half of the studies did not adjust for both stratification variable(s) and clustering. Moreover, almost half of the studies adjusted the primary method for clustering only, while this number varies from 37 to 92% in the previous studies on the review of CRTs [2, 16–24].

Along with performing the adjusted analyses, we also need to focus on other areas of stratified cluster randomization trials including sample size calculation and randomization. Similar to the randomized controlled trials on individuals, it is necessary to report all the information used to calculate the sample size, including detectable difference, level of significance, and desirable power. Most importantly, sample size calculation needs to account for the clustering. In this survey, we have found 62% of the studies reported the ICC/CV used to calculate the sample size or power. This number varies from 0 to 71% in the previous reviews on CRTs [2, 16–24]. Furthermore, it is also necessary to report the randomization method used to allocate clusters to intervention groups within each stratum—which was not reported by more than half of the studies in this survey.

There were significant deficiencies in reporting the results from the stratified CRT. Reporting on the following areas, at minimum, would better represent and help the audience to better understand the stratified nature of this type of studies: (1) only a few studies provided the reasoning for stratification. Reporting the reasoning for stratified design and choosing the stratification variable(s) would be helpful; (2) more than 20% of the studies did not provide the definition of all the strata. For a stratified design, it is essential to report how all the strata are defined; (3) almost all the studies provided the study flow chart, while only 19% and 31% of the studies included stratification variables/strata in the flow chart or in baseline characteristics table, respectively. Inclusion of stratification variables in the flow chart or baseline characteristics table would provide the clear depiction of the design; (4) only 20% and 11% of the studies reported the stratum-specific number of clusters and individuals in the intervention groups, respectively. Thus, more attention is needed to report these numbers; (5) reporting the stratum-specific, if possible, would help the readers to know the intervention effect in each stratum.

The major strength of this study was that we used the search terms recommended by Taljaard et al. [27] to select the stratified cluster randomized trials from one of the largest database MEDLINE. Also, we included the published main trial results of the protocols selected in title and abstract screening. Furthermore, this survey was based on the time period from 1946 to 2019. The major limitation of this study is that only one reviewer conducted this survey. Despite multiple checking or best effort, it is possible that the reviewer may have failed to include some of the eligible studies.

A well-designed large-scale systematic review would depict a more complete picture about the analysis and reporting status of stratified cluster randomized trials. Following the CONSORT extension for CRTs [26] or inclusion of a statistician [24] in the study will significantly improve the quality of reporting and analysis of stratified CRTs. Furthermore, CONSORT statements for CRTs can be extended for stratified CRTs, incorporating the recommendations we made in this manuscript. This type of extension would be helpful for the researchers as it will guide them about the analysis and reporting of stratified cluster randomized trials.

## Conclusion

In this systematic survey, we assessed the current practice about reporting and analysis of stratified cluster randomized trials. We found that there were substantial deficiencies in the reporting of methodological aspects of stratified cluster randomized trials. Furthermore, majority of the studies did not adjust their primary method of analysis for both clustering and stratification—which were important to assess the intervention effect for stratified cluster randomized trials. Moreover, stratified cluster randomized trials require substantial improvement in reporting such as details about sample size calculation and randomization, definition of all strata, inclusion of stratification variable(s)/strata in study flow chart or baseline characteristics table, and stratum-specific number of clusters and individuals in the intervention groups. A guideline would be helpful for researchers to enhance the transparent reporting and analysis of stratified cluster randomized trials.

## Supplementary Information

**Additional file 1.** PRISMA 2009 Checklist.
